# Elevation, Not Deforestation, Promotes Genetic Differentiation in a Pioneer Tropical Tree

**DOI:** 10.1371/journal.pone.0156694

**Published:** 2016-06-09

**Authors:** Antonio R. Castilla, Nathaniel Pope, Rodolfo Jaffé, Shalene Jha

**Affiliations:** 1 Department of Integrative Biology, 401 Biological Laboratories, University of Texas, Austin, TX, 78712, United States of America; 2 Vale Institute of Technology–Sustainable Development, Rua Boaventura da Silva 955, Belém, PA 66055–090, Brazil; 3 Department of Ecology, University of São Paulo, Rua do Matão 321, São Paulo, SP 05508–090, Brazil; Chinese Academy of Forestry, CHINA

## Abstract

The regeneration of disturbed forest is an essential part of tropical forest ecology, both with respect to natural disturbance regimes and large-scale human-mediated logging, grazing, and agriculture. Pioneer tree species are critical for facilitating the transition from deforested land to secondary forest because they stabilize terrain and enhance connectivity between forest fragments by increasing matrix permeability and initiating disperser community assembly. Despite the ecological importance of early successional species, little is known about their ability to maintain gene flow across deforested landscapes. Utilizing highly polymorphic microsatellite markers, we examined patterns of genetic diversity and differentiation for the pioneer understory tree *Miconia affinis* across the Isthmus of Panama. Furthermore, we investigated the impact of geographic distance, forest cover, and elevation on genetic differentiation among populations using circuit theory and regression modeling within a landscape genetics framework. We report marked differences in historical and contemporary migration rates and moderately high levels of genetic differentiation in *M*. *affinis* populations across the Isthmus of Panama. Genetic differentiation increased significantly with elevation and geographic distance among populations; however, we did not find that forest cover enhanced or reduced genetic differentiation in the study region. Overall, our results reveal strong dispersal for *M*. *affinis* across human-altered landscapes, highlighting the potential use of this species for reforestation in tropical regions. Additionally, this study demonstrates the importance of considering topography when designing programs aimed at conserving genetic diversity within degraded tropical landscapes.

## Introduction

Forest ecosystem function is dependent on the colonization and establishment of native trees, which provide essential ecosystem services such as carbon sequestration, erosion control and the provisioning of food and nesting resources for humans and wildlife [1]. However, many of the world’s forests are threatened by frequent and intense human-driven disturbance events, and rates of land conversion to agriculture, pasture, and urban areas are expected to further increase in response to a growing human population [2]. As vast regions of primary forest have already been cleared, recent conservation efforts are emphasizing the reforestation of degraded habitats as an additional strategy to safeguard ecosystem services in key areas of ecological and economic importance [3,4]. Specifically, there is growing interest from both conservation managers and landholders in the use of early successional native tree species for reforestation instead of fast-growing exotic timber species, as these native species can have beneficial impacts on both biodiversity conservation and rural livelihoods [5,6].

Reforestation implies the release of cultivated and translocated trees, which can have important genetic consequences for native populations such as alterations in their genetic diversity and gene flow dynamics and breakdowns of local adaptation [7–9]. Thus the design of effective restoration strategies using native trees requires detailed knowledge of genetic diversity and structuring within and among native populations of the selected species [10,11]. A first step in reforestation programs must therefore be the screening of native population genetic structure and levels of genetic diversity prior to implementation [10]. Furthermore, the successful establishment of translocated trees may depend on the species’ breeding system (self- vs. out-crossed), the impact of inbreeding on the offspring, and the ongoing gene flow from native source populations [12]. Thus, understanding how native pioneer species have colonized areas in the past and how their genetic diversity is currently structured across the landscape is critical for the design of reforestation strategies within degraded areas.

The first and simplest hypothesis regarding gene flow across space is the isolation by distance pattern (IBD, hereafter). The IBD pattern is characterized by a declining probability of genetic identity with geographic distance [13] and has been reported for many living organisms, existing largely as a result of drift-dispersal processes (reviewed in [14]). Within the context of conservation genetics, IBD may suggest that optimal conservation of genetic diversity and potential adaptive genetic variation will require conservation of multiple populations across the species range [15,16]. Furthermore, IBD has frequently been reported for trees (e.g. [17,18]), although there are exceptions to this rule (e.g. [19]). Specifically, in some cases, topographic or geographic barriers, such as mountain ranges, may better explain divergence in genetic composition than geographic distance alone (e.g. [19]).

Thus, a second potential factor influencing gene flow among tree populations is elevation. As in any ecological gradient, many species have an ecological optimum along elevational gradients [20,21]. For instance, lowland plant species typically exhibit abundance peaks at low elevations and declines towards upper altitudinal limits as a consequence of suboptimal habitats. In this context, pollinators and frugivores can shift along elevational gradients to other more abundant resources, potentially compromising the dispersal service to lowland species at high elevation sites [22,23]. Furthermore, elevational gradients drive variation in environmental factors such as temperature or precipitation [24], which may affect flying conditions for insects, and potentially impact pollination [25–27]. Variation in the abiotic environment along elevational gradients may also lead to marked differences in the phenology of both flowering and fruiting events, also potentially altering pollination and seed dispersal [28,29]. Specifically, recent studies have suggested that elevation-mediated phenological time lags can impact both migration and selfing rates in tree species, potentially leading to long-term genetic structure across elevation gradients [30]. While a number of studies have found evidence of genetic differentiation for plant species living along elevational gradients [31], this past work has largely focused on small spatial scales (1–10km) and has not explicitly investigated species with short generation times and early-successional life history strategies.

Finally, forest fragmentation is expected to reduce genetic diversity, increase genetic differentiation, and potentially increase inbreeding in tree populations [32,33]. However, the empirical support to these theoretical expectation remains elusive [34,35]. A number of studies have indicated that the response of each species to habitat loss may differ greatly depending on their biological attributes, in particular animal vs. wind dispersal [36,37]. For instance, some tree species are able to ameliorate the genetic consequences of fragmentation through flexible mating systems and animal dispersers with high mobility [38–40]. For other tree species, though forest fragmentation may alter ecological processes, alterations may be challenging to detect if the process of fragmentation has occurred over a shorter period relative to the generation time of the species [35]. Therefore, it is important to examine trees of younger age-classes or progenies sired in those landscapes where deforestation has been recent [35].

Given the potentially major impacts of distance, elevation, and forest cover on plant gene flow, it is particularly urgent to investigate gene flow processes for tropical trees, as tropical regions exhibit substantial elevation gradients, greater proportions of animal-dispersed plant species, and rapid rates of deforestation. Specifically, species ranges in the tropics are frequently delimited by elevation, with distinct community assembly processes along elevation gradients [41] and potential implications for elevation-mediated gene flow. Second, most tropical tree species are animal-pollinated and self-incompatible and thus dependent on mobile animals for reproduction, making them particularly vulnerable to declines in disperser communities [42]. Finally, tropical biomes have the highest deforestation rates of any other biome, with an estimated ~2100 square kilometers of tropical forest destroyed every year [43]. Despite these factors, we still know very little about how tropical landscape composition and structure impacts plant dispersal and gene flow.

In this study, we use landscape genetic tools to assess how geographic distance, elevational gradients, and forest cover influence patterns of genetic structure and genetic diversity in a pioneer tree species across the Isthmus of Panama. This region is a global biodiversity hotspot that suffers from deforestation, biodiversity loss, and heavy erosion in streams and canals [44,45] and thus there is great need to address the potential of early successional native tree species for restoration programs. Our study model is *Miconia affinis*, a common early successional Neotropical tree that frequently colonizes forest gaps, riparian areas, and exposed hillsides [46]. *Miconia* represents one of the most abundant and diversified genera in the Neotropics [47,48], and because *M*. *affinis* is dispersed by a diverse pollinator and seed disperser community [46,49], it may be a particularly critical species for facilitating pollinator and frugivore community assembly in degraded regions. Specifically, we address how deforestation, elevation, and geographic distance influence the genetic diversity and differentiation of *M affinis* populations, and how gene flow has changed across historic and contemporary time periods. By examining genetic diversity, genetic differentiation, and migration rates within human-altered regions, this study determines the ability of a native species to maintain effective dispersal and colonization across both pristine and disturbed tropical landscapes. Further, we provide management suggestions that may be relevant for the many tree species with similar phenology and life histories.

## Materials and Methods

### Study species

*Miconia affinis* D.C. (Melastomataceae) is a common, early successional self-incompatible understory tree (3–6 m height) that is broadly distributed in the Neotropics, ranging from Mexico to Brazil [49,50]. The species blooms for ≈ 2 days at the onset of the first rains (January to June), and its flowers are visited by a large diversity of social and solitary bees [50]. Fruits develop in 3–4 months (May-July) and seeds are dispersed by a variety of birds and bats ([46] and references therein).

Previous studies have reported short lifespans (i.e., < 20 years) and early first flowering events (i.e., 4–5 years) for several *Miconia* species of similar size and habit [51–53]. To confirm this life history strategy for *M*. *affinis*, we estimated the annual growth rate of the species using data from the 50-ha Forest Dynamic Plot on Barro Colorado Island (BCI), Panama [54–56]. The plot consists of a standing number of over 350,000 mapped stems 10mm or above in diameter at breast height (DBH) and approx. 300 plant species (http://ctfs.arnarb.harvard.edu/webatlas/datasets/bci/). Censuses have been conducted since 1982. Specifically, we used data from the censuses conducted from 1990 to 2010 (http://www.ctfs.si.edu/site/Barro+Colorado+Island/abudance/) for trees that survived the whole 20-year period, as this was the period when trees were censused every 5 years (N = 32 trees)[57]. We estimated the annual growth rate as per Condit and colleagues [58], a method widely used for tropical tree species (e.g. [59]). For each tree, we estimated the annual growth rate as the difference in DBH between 2010 and 1990 divided by the number of years (i.e., 20 years). We posit that early growth in *M*. *affinis* (i.e., seedling and sapling stages) would not substantially alter our growth estimates given that congeneric species (e.g. *M*. *argentea)* exhibit growth from germination to the 1m tall size class in 1 to 6 years [60].

**Fig 1:**
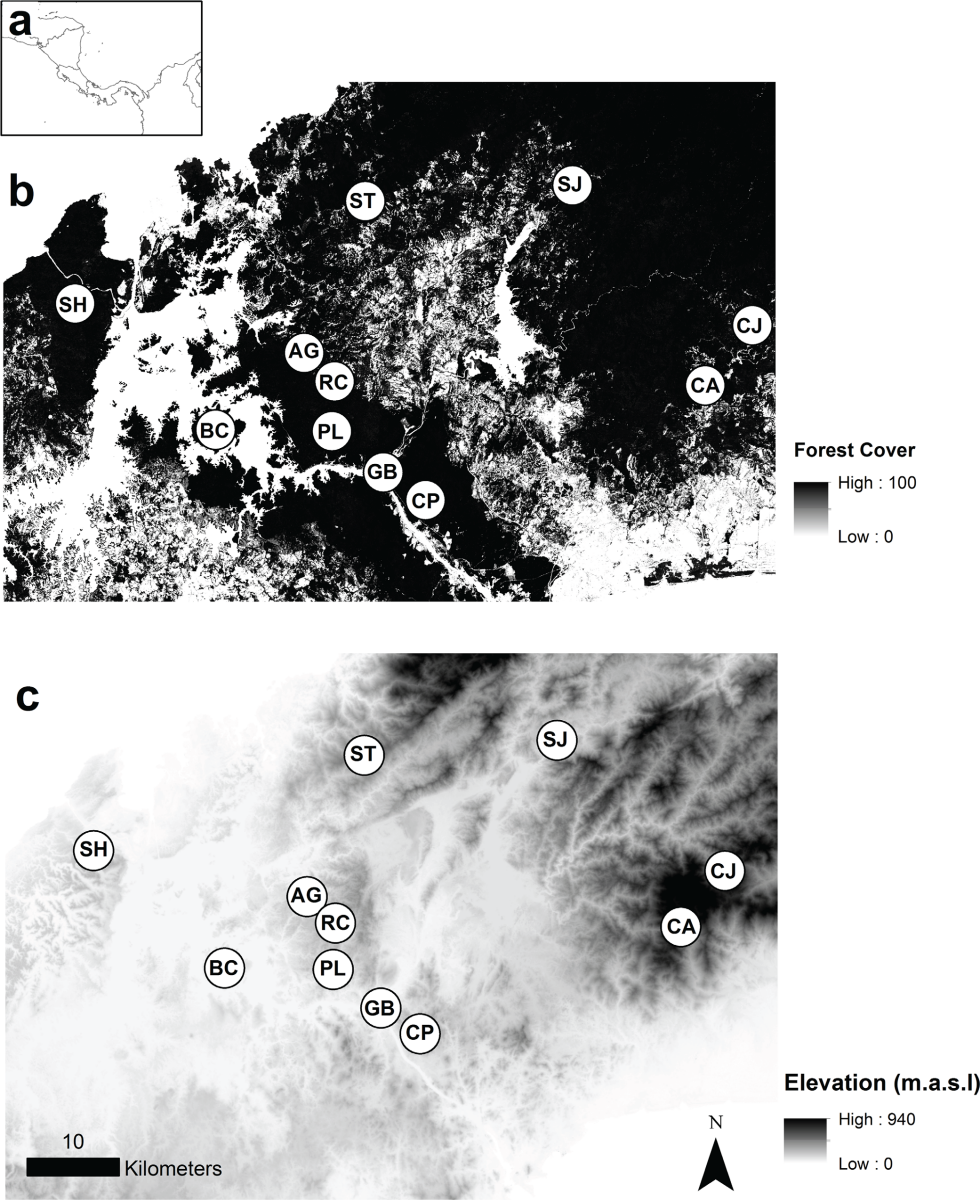
Study region. (a) Location of the study in Central America and (b & c) distribution of *M*. *affinis* populations across the Isthmus of Panama. (b) Forest cover, where increasing percentages of forest cover are represented by darker shades (0–100%) and (c) elevation, where increasing elevation is represented by darker shades (0–940 m.a.s.l.), and for both cases, darker shades represent higher resistance habitat. The location of the study populations in the map corresponds with the population centroids.

Using this information, we estimated the lifespan of the species by calculating the approximate number of years to reach the largest DBH. Specifically, we used data from five populations along the Panama Canal (CP, GB, PL, AG and RC; Fig 1) in which we surveyed all *M*. *affinis* trees ≥ 10 mm DBH in a plot of 3 km x 3 km per population (358, 349, 96, 316 and 370 individuals respectively). Using the largest DBH in each population, we calculated the mean largest DBH for *M*. *affinis*. We estimated the lifespan (LS) by dividing the mean largest DBH by the mean annual growth rate.

We also estimated the generation time (T_g_) as the age at which members of a given cohort are expected to reproduce. Based on previous studies of the species, only *M*. *affinis* trees with diameter at breast height ≥ 10 mm produce flowers [61]. Therefore, we estimated the generation time (T_g_) in years by dividing the DBH when the tree reproduces for the first time (i.e., 10 mm) by the mean annual growth rate.

### Study region

This study was conducted across the Panama Canal bioregion, an area that has lost more than 30% of its forested area to agro-pastoral development during the past 50 years [45]. We received permission for our field work and collection of samples from Panama’s National Authority for the Environment (ANAM). Using reported localities for *M*. *affinis* from the University of Panama’s Herbarium, we extensively surveyed the Isthmus of Panama locating eleven populations (Fig 1A–1C). The elevation of our study sites ranged from 26 to 804 m.a.s.l. (mean = 257.5 m.a.s.l.; Table 1), and the geographic distance between populations ranged from 4.2 to 67.9 km (mean = 28.5 km).

**Table 1:** Attributes of *M*. *affinis* populations. Population (Pop.) geographic coordinates (Lat. and Long.), elevation, number of trees (N), mean diameter at breast height (DBH), allelic richness (AR), private allelic richness estimated by rarefaction (PAR), and Nei’s gene diversity (He).

Pop	Lat.	Long.	Elevation (m.a.s.l.)	N	DBH (mm)	AR	PAR	Unbiased He
					Mean (SD)	Mean (SD)	Mean (SD)	Mean (SD)
AG	9.2256	-79.766	120	29	27.85 (11.89)	3.83 (0.57)	0.19 (0.09)	0.635 (0.087)
BC	9.1554	-79.847	150	30	44.23 (21.30)	3.76 (0.46)	0.07 (0.03)	0.631 (0.075)
CA	9.1958	-79.400	628	30	53.60 (20.29)	2.92 (0.46)	0.08 (0.05)	0.505 (0.098)
CJ	9.2509	-79.357	804	30	48.53 (17.86)	3.78 (0.44)	0.23 (0.06)	0.616 (0.078)
CP	9.0910	-79.655	164	30	48.38 (19.89)	3.38 (0.46)	0.15 (0.07)	0.609 (0.066)
GB	9.1163	-79.694	26	28	43.31 (16.53)	3.68 (0.52)	0.12 (0.05)	0.616 (0.081)
PL	9.1540	-79.741	40	30	38.71 (12.95)	3.77 (0.56)	0.15 (0.06)	0.620 (0.093)
RC	9.1995	-79.738	183	30	32.64 (11.72)	3.42 (0.53)	0.08 (0.04)	0.597 (0.090)
SH	9.2706	-79.975	180	30	49.17 (20.74)	3.66 (0.63)	0.18 (0.09)	0.571 (0.100)
SJ	9.3787	-79.521	98	30	29.52 (13.07)	3.82 (0.59)	0.15 (0.07)	0.620 (0.104)
ST	9.3642	-79.710	440	25	35.47 (17.16)	3.53 (0.64)	0.19 (0.09)	0.563 (0.121)
*Mean*					*41*.*13 (18*.*76)*	*3*.*60 (0*.*08)*	*0*.*14 (0*.*02)*	*0*.*598(0*.*012)*

We assessed the influence of elevation on genetic structure using a digital elevation map (DEM) of the study region with a resolution of 30 m x 30 m, available through STRI GIS online portal (http://strimaps.si.edu/portal/home/index.html). We examined the influence of contemporary deforestation patterns by utilizing a high-resolution global tree canopy cover map for the year 2000 with a resolution of 30 m x 30 m (University of Maryland: http://earthenginepartners.appspot.com/science-2013-global-forest). Forest cover represents the percentage of canopy closure for all vegetation taller than 5 meters in height, and each pixel is encoded with the % forest cover value, ranging from 0 to 100% in the study region (mean forest cover = 63.14%). In addition, we analyzed the influence of mean annual precipitation and mean annual temperature using the Worldclim database (http://www.worldclim.org/), at a resolution of 2.5 arc-min (roughly 5 km at the equator).

### Sampling and genotyping

We collected leaf tissue from 25–30 randomly chosen trees bearing inflorescences or infructescences (reproductive trees, hereafter) in each of the 11 populations (mean = 29 trees per population, total of 322 trees; Table 1). Tissue was preserved in liquid nitrogen and stored at -80°C until genotyping. Total genomic DNA was extracted from adult leaf tissue using the CTAB protocol [62]. All trees were genotyped for a set of nine highly polymorphic microsatellite loci [63,64]. Multiplex polymerase chain reactions (PCRs) using fluorescent dye-labeled selective primers were performed using a QIAGEN multiplex PCR kit (QIAGEN). Fragment separation and detection were conducted using an ABI 3730 DNA Sequencer, and alleles were scored manually using GENEMARKER® (Softgenetics).

### Allelic richness, gene diversity, and population structure

The probability of null alleles was calculated using the software Micro-Checker [65], and deviations from HWE and linkage disequilibrium (LD) were tested in GENEPOP v 4.0.10 [66] with 1000 dememorizations, 100 batches, and 1000 iterations per batch using the Markov chain approximation for the exact tests and likelihood-ratio tests, respectively. As Micro-Checker results indicated that one locus (B102) exhibited substantial evidence of null alleles (>70% of populations with evidence of null alleles), we chose to exclude this locus from subsequent analyses. One locus (Micaff 19) showed strong evidence of LD, so we also excluded it from subsequent analyses. The remaining seven loci exhibited deviations from HWE in less than 50% of populations, so we kept these seven loci for subsequent analyses.

Allelic richness (AR) and private allelic richness (PAR) per population were estimated using rarefaction with 10 genes as the sample size in HP-RARE [67], and unbiased Nei’s gene diversity was calculated using GenAlEx 6.501 [68]. For each population, we calculated the geographic distance to the ten other populations (spatial isolation, hereafter) and the percentage of forest cover within a 3 km radius. We analyzed the effect of spatial isolation, elevation, and forest cover on our estimates of genetic diversity (i.e., AR, PAR and gene diversity) using generalized linear models. For each response variable, we built 7 a priori models comprised of various combinations of the three explanatory variables (spatial isolation, elevation, and forest cover) and used Akaike information criterion (hereafter, AIC) as a criterion for model selection. For the three explanatory variables, we estimated the model averaged parameter estimates (β) with standard errors based on models with ΔAIC of less than 5, using the package MuMIn for R [69].

A two-level hierarchical analysis of molecular variance (AMOVA; [70]) was run to analyze the partitioning of molecular variance among and within populations, using the program GenAlEx 6.501 [68]. We examined population genetic structuring using the Bayesian clustering method in STRUCTURE 2.3.3 [71]. The number of genetic groups was explored by performing 10 replicates of each simulation from K = 1 to K = 11, with a burn-in of 50,000 and MCMC of 100,000 assuming admixture and correlated frequencies [71]. We implemented CLUMPP using Structure Harvester [72] and we applied modal ΔK parameter as the choice criterion [73] to detect the true number of genetic groups. As a follow-up, we ran additional AMOVAs on the clusters assigned from the STRUCTURE analyses to compare the partitioning of variance among and within the assigned clusters.

### Landscape genetic analysis

Using circuit theory [74], we examined if genetic differentiation among *M*. *affinis* populations is accounted for by three explanatory variables: geographic distance, elevation, and deforestation. We estimated the mean effective resistance between all pairs of sample sites (pairwise mode in the Circuitscape software; [74]) and define these values as Geographic Resistance Distance (RD), Elevation RD, and Deforestation RD. This software is based on electronic circuit theory and evaluates contributions of multiple dispersal pathways [74]. Geographic RD was analyzed by running a raster with equal resistances for all pixels. We did this by assigning all pixels a 0.5 resistance and then calculating resistance distances. Null resistance distances were log-transformed. Deforestation represents the percentage of open canopy for all vegetation taller than 5 meters in height; each pixel was encoded with the % deforestation, which we obtained by subtracting its forest cover percentage from 100%. Elevation ranged from 0 to 940 m.a.s.l. in the study region, and each pixel was encoded with the raw elevation value before resistance distance was calculated. Zero values in the rasters were replaced by 0.0001, since Circuitscape does not accept zeros. Finally, mean annual temperature and mean annual precipitation ranged from 22.3°C to 26.9°C and from 1754 mm to 3812 mm respectively in the study region, and each pixel was encoded with the raw environmental value before resistance distances were calculated.

We used maximum likelihood population effects (MLPE) models to test for the relationship between pairwise genetic distance [F_ST_ / (1-F_ST_)] and pairwise explanatory variables [75]. We also ran the same models using G_ST_ and D_ST_ instead of F_ST_ as estimates of the genetic differentiation among *M*. *affinis* populations. We assessed multi-collinearity between predictors before conducting our analyses using the variance inflation factor (VIF function, package car; [76]) and this prevented the incorporation of additional predictors such as Precipitation RD and Temperature RD, both of which correlated with Geographic RD and exhibited a VIF > 10. Among these three factors, we chose to include Geographic RD because the isolation by distance pattern is the most parsimonious explanation for genetic differentiation among plant populations [14]. Furthermore, elevation is often a driver of temperature and precipitation patterns [24], and thus inclusion of the Elevation RD allows us to indirectly explore the role of precipitation and temperature (see S1 Fig).

Overall, we examined a set of three explanatory variables, Geographic RD, Elevation RD, and Deforestation RD in our MLPE models (VIF < 2 for all variables). In our MLPE models, genetic distance was used as the response variable and resistance distances as predictors. We centered all explanatory variables around their mean and fitted MLPE models with Maximum Likelihood estimation using the “gls” function in the “nlme” R package [77]. The MLPE model uses a residual covariance structure to account for the non-independence of pairwise distances and is becoming a standard approach in landscape genetic studies since it accounts for the non-independence of pairwise distances within a likelihood framework and is compatible with AIC-based model selection [78]. Code implementing the MLPE correlation structure within the R package nlme is provided [79,80]. We built 7 a priori models comprised of various combinations of the three explanatory variables (geographic distance, elevation, and deforestation). For the three explanatory variables, we estimated the model averaged parameter estimates (β) with standard errors based on models with ΔAIC of less than 5 using the package MuMIn for R [69]. Finally, some studies have reported an increased abundance of pioneer tropical trees and resilient gene flow among their populations with forest fragmentation [81]. For this reason, we repeated our landscape genetic analysis running the same MLPE models but coding Deforestation as a conductance variable (i.e., Deforestation CD) and results were consistent using both approaches. Finally, we also ran the same MLPE models but used raw elevation differences between populations instead of Elevation RD.

### Historical and contemporary gene flow

To compare migration rates over contemporary and historical timescales, we used the programs BAYESASS and MIGRATE [82,83], respectively. Both programs generate parameters from which a comparable measure of gene flow can be inferred (*m*: proportion of population consisting of genetic migrants per generation). However, each program estimates this parameter over a different timescale. BAYESASS uses a Bayesian approach with MCMC sampling to estimate recent migration rates over the last five to seven generations (i.e., 25–35 years) given a generation time of 5 years for *M*. *affinis* (i.e., time to the first flowering event). This method does not assume the population to be in HWE or migration-drift equilibrium. The program was run for 5 x 10^6^ iterations of which 500,000 were burn-in. We performed 3 runs with different seed numbers to ensure the convergence and consistency among runs. We used MIGRATE-n v. 3.6.11 to estimate levels of historical gene flow (approx. 4*N*_e_ generations in the past, approximately 1000’s of years) and effective population size. MIGRATE uses a coalescent method with MCMC to estimate the mutation-scaled effective population size (*θ* = 4*N*_e_μ, where *N*_e_ is effective population size and μ is mutation rate) and the mutation-scaled migration rate (*M* = *m*/μ). All starting values were optimized by multiple test runs, adjusted for subsequent runs. In the final run, we used 10 short chains of 500,000 trees sampled and 5,000 trees recorded and 4 long chains of 5,000,000 trees sampled and 50,000 recorded. We used four heated chains with temperatures, 1.0, 1.5, 3.0 and 100,000.0, to ensure sufficient sampling of genealogical space. MIGRATE assumes that populations are in migration-drift equilibrium but does not require them to be in HWE.

To compare historical and contemporary migration rates, we used the values of *m* directly generated by BAYESASS and the estimated *m* from values of *M* (m/μ) generated by MIGRATE by dividing all *M* values by an estimated mutation rate of 5 x 10^−4^ [84]. Concordance of historical and contemporary migration rates was checked using a Mantel test with 10000 permutations using the function “mantel” in the “ecodist” R package [85].

### Test of population history

Finally, we evaluated which of two models of demographic history (gene flow versus genetic drift only) best described processes leading to the current genetic structure in the *M*. *affinis* populations using the approach developed by Ciofi and colleagues [86] and implemented in the program 2MOD by MA Beaumont (software available from http://www.maths.bris.ac.uk/~mamab/software/). This program assesses the relative likelihoods of both demographic models using MCMC procedure. The gene flow model assumes populations are at drift-migration equilibrium and uses the allelic frequencies within populations to estimate the relative strength of drift versus gene flow for each population. The drift model assumes a historical panmictic population separated into many smaller populations that have since been evolving independently through genetic drift alone in the absence of gene flow. Both models assume that mutation rate is small and calculate a parameter F, the probability that two alleles in a given population share a common ancestor. We ran the program twice for each model with 500,000 MCMC updates, discarding the first 10% as burn-in. Results from the two runs were combined and the probability of each model was calculated as the number of draws for a given model out of the total draws.

## Results

### Lifespan and generation time of *M*. *affinis*

We found a mean annual growth rate of 1.75 ± 0.16 mm per year for *M*. *affinis* in the 50-ha BCI plot. Based on these data, we predict an average lifespan of 64.3 years and a generation time of 5.71 years for *M*. *affinis* (S1 Table). According to this estimated growth rate, 50 year-old trees would exhibit DBH > 87.5 mm. In the five intensively surveyed populations in this study (CP, GB, PL, AG and RC), the DBH ranged from 7.55 to 125.5 mm with a mean value of 37.29 mm. In our data set, only 2% of trees have DBH greater than 87.5 (older than ~50 years), 31% have DBH ranging from 43.75 to 87.5 (~ 25–50 year-old), while 67% of trees have DBH < 43.75 mm (i.e., 1–25 year-old).

### Allelic richness, gene diversity, and population structure

Average allelic richness per site based on rarefaction was 3.60 (±0.08) and average private allelic richness per site was 0.14 (±0.02). Average within-population unbiased Nei’s gene diversity was 0.598 (±0.027) (Table 1). Neither spatial isolation, nor elevation were related to private allelic richness (*P* = 0.41 and *P* = 0.49 respectively). There was a marginally significant positive relationship between private allelic richness and forest cover (*z* = 1.86, *P* = 0.06). Allelic richness was not related to spatial isolation, elevation, or forest cover (*P* = 0.85, *P* = 0.27 and *P* = 0.90, respectively). The unbiased gene diversity was also not related to spatial isolation, elevation, or forest cover (*P* = 0.28, *P* = 0.13 and *P* = 0.69 respectively). We also ran the same models using the percentage of forest cover within a smaller 2 km and 1 km radius and obtained consistent results (see S2, S3 and S4 Tables in Supporting Information).

**Fig 2:**
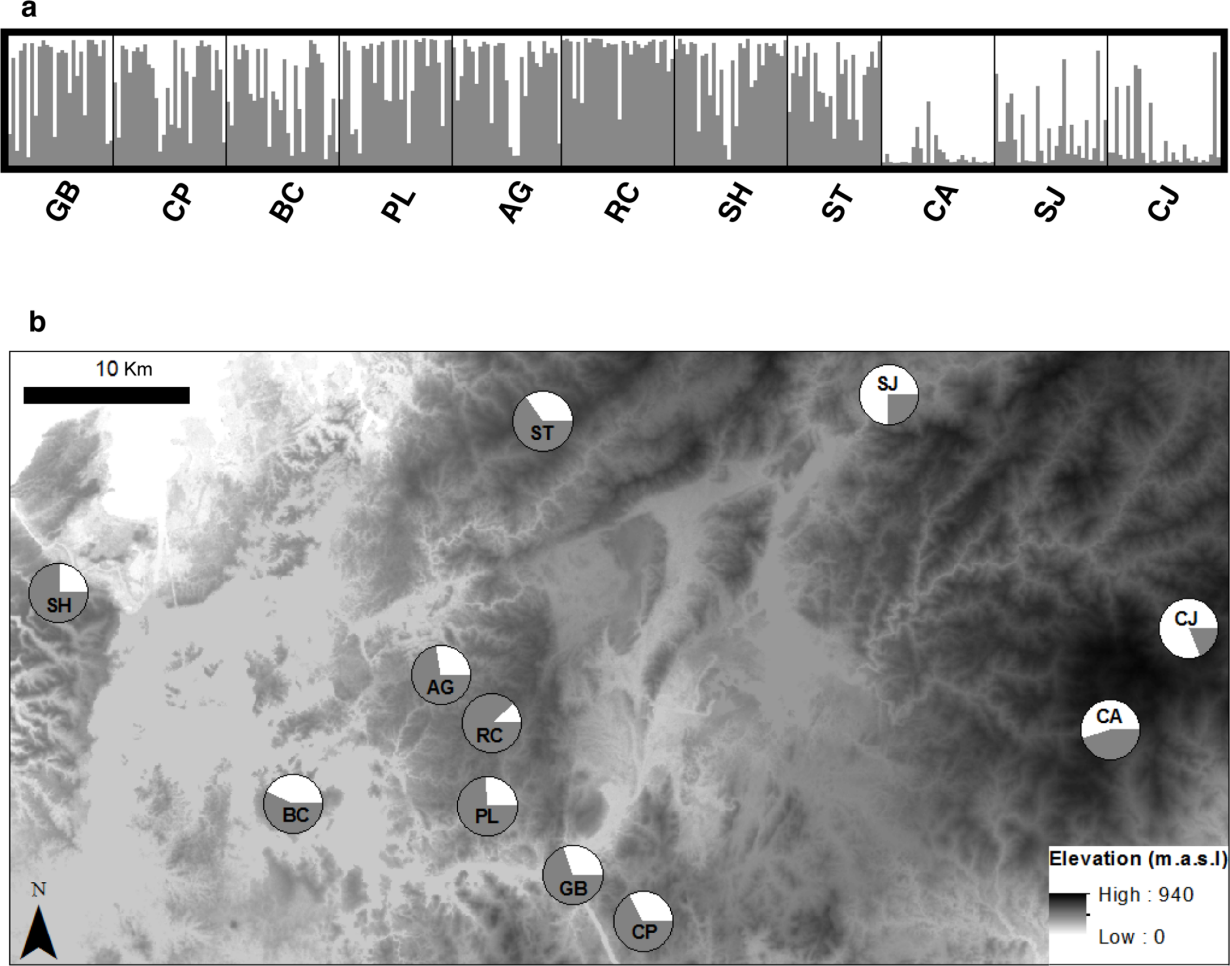
Genetic structure of *M*. *affinis* populations based on STRUCTURE output for K = 2. (a) Structure bar plot with individuals sorted by populations. Each vertical bar represents a single individual and color segments are proportional to its membership in two genetic clusters inferred from STRUCTURE analyses. From left to right, populations are sorted by increasing distance to the Panama Canal. (b) Populations represented by individual pie charts with the mean proportion of membership of each population for the inferred number of K = 2 genetic groups. In the background, higher elevation is represented by darker shades (0–940 m.a.s.l.).

AMOVA revealed significant genetic differentiation among populations (F_ST_ = 0.08, P < 0.001), with 8% of molecular variance accounted for by differences among populations. Results of the STRUCTURE analysis revealed a modal maximum of ΔK at K = 2, although it was only two times higher than the ΔK at K = 3 (S2 Fig). At both values of K, the level of admixture was substantial (Fig 2 and S3 Fig). At K = 2, three eastern populations (i.e., CA, CJ and SJ) were differentiated from the remaining populations (Fig 2A and 2B). At K = 3, our results showed an additional third group consisting of ST, SH, and BC populations (S3 Fig). For K = 2 and K = 3, AMOVAs conducted on the STRUCTURE assigned clusters revealed 7% of the variation was due to differences among clusters in both analyses.

### Landscape genetic analysis

**Fig 3:**
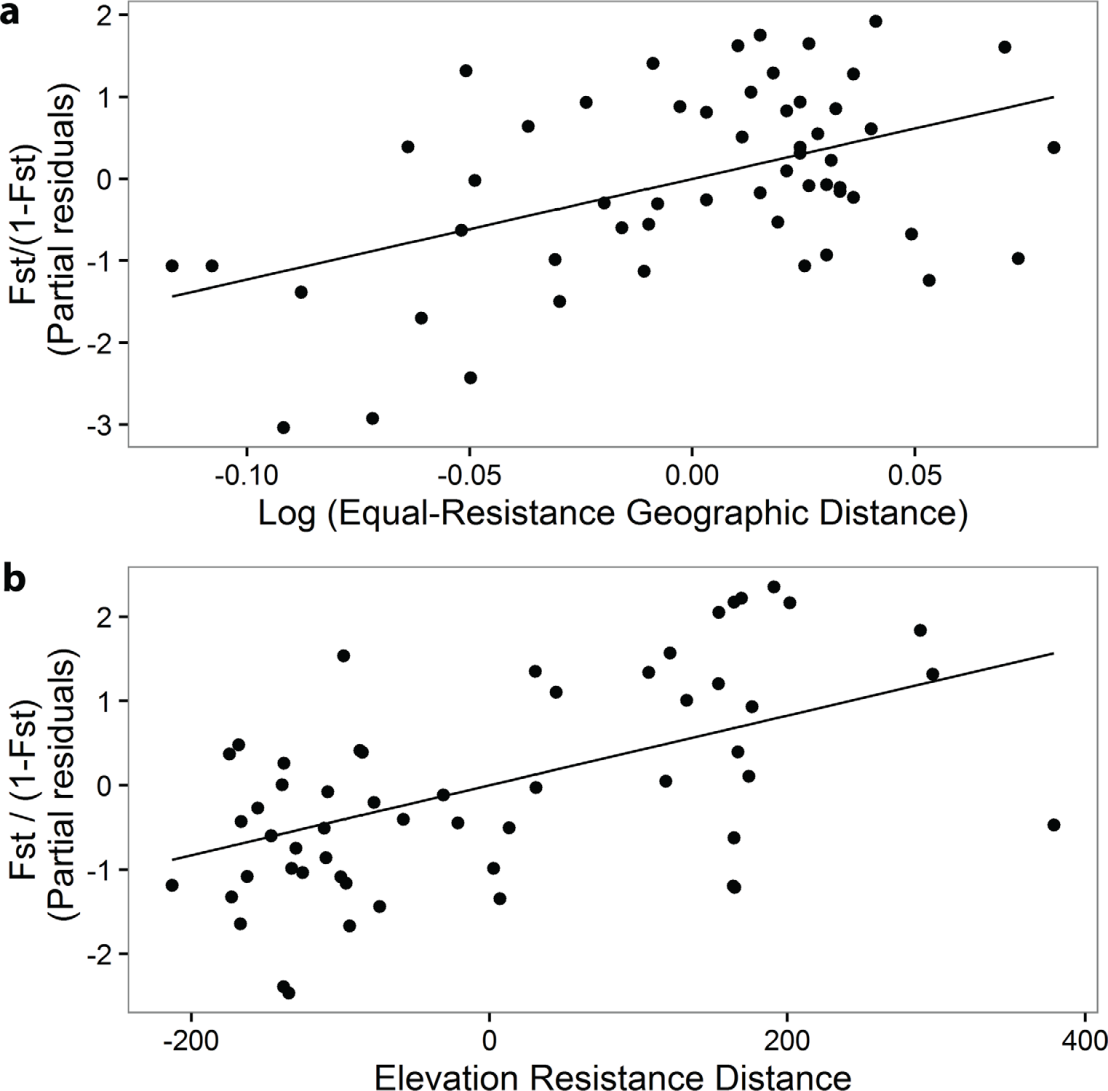
Isolation by Resistance Distance. (a) Isolation by Geographic Resistance Distance (RD) and (b) Elevation RD. Pairwise comparisons of genetic differentiation (F_ST_) as a function of (a) Geographic RD and (b) Elevation RD. Explanatory variables are centered around their mean. We graphically represent the partial residuals of the dependent variable to remove the effects of other explanatory variables included in the model. Partial residuals were computed using the top-ranked model (ΔAIC; see Table 2).

The first ranked model contained the explanatory variables Geographic RD and Elevation RD, with a 59% probability of being the best approximating model in the set (Table 2). Our results showed a positive relationship between genetic distance and Geographic RD (Table 3, Fig 3A). Our results also supported an isolation by elevation pattern with a positive relationship between the genetic distance and the Elevation RD (Table 3, Fig 3B). We obtained consistent results using G_ST_ and D_ST_ as response variables instead of F_ST_ and using raw elevation differences between populations instead of Elevation RD (S5–S10 Tables).

**Table 2:** Ranked models explaining landscape effects on genetic differentiation (i.e., F_ST_) among *M*. *affinis*’s populations. Models are ranked based upon the difference between Akaike’s Information Criterion (AIC) in each individual model and the lowest AIC model (ΔAIC). Akaike’s weights (ω_i_) for each model are listed. The genetic differentiation between populations was calculated as [F_ST_ / (1-F_ST_)] and Geographic refers to Geographic Resistance Distance (RD), Deforestation refers to Deforestation RD, and Elevation refers to Elevation RD.

Model statement	AIC	ΔAIC	ω_i_
Geographic + Elevation	-240.66	0.00	0.588
Geographic + Elevation + Deforestation	-238.93	1.73	0.247
Geographic	-237.49	3.17	0.120
Geographic + Deforestation	-235.51	5.15	0.045
Elevation	-225.49	15.17	0.000
Elevation + Deforestation	-224.00	16.66	0.000
Deforestation	-216.16	24.50	0.000

**Table 3:** Model averaged coefficients (β) and their standard errors (SE) calculated from the candidate model set (i.e. models with ΔAIC < 5). The genetic differentiation between populations was calculated as [F_ST_ / (1-F_ST_)]. Geographic refers to Geographic Resistance Distance (RD), Deforestation refers to Deforestation RD, and Elevation refers to Elevation RD.

	β	SE	Z value	P value
Geographic	0.40800	0.09091	4.390	<0.001*
Elevation	0.00013	0.00006	2.362	0.018*
Deforestation	-0.00144	0.00283	0.495	0.621

* Model averaged coefficients not overlapping with zero

### Historical and contemporary gene flow

**Fig 4:**
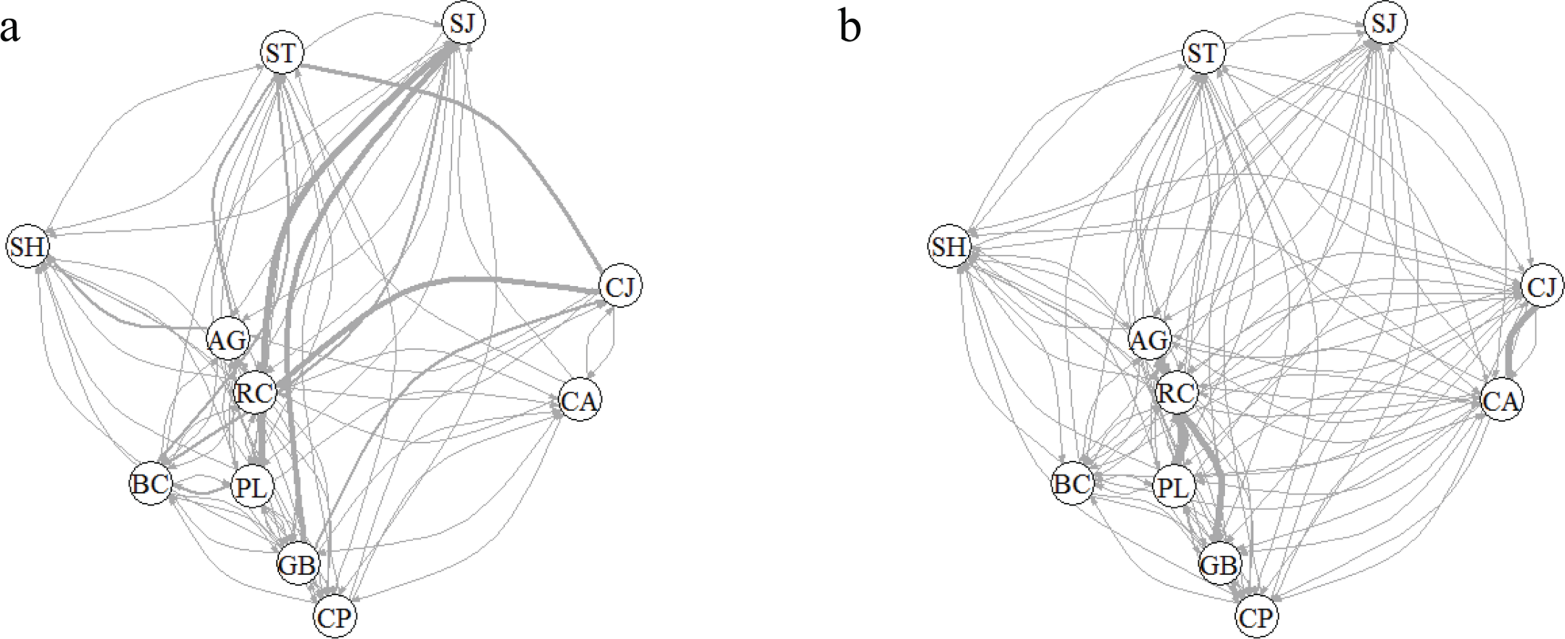
Historical and contemporary migration among *M*. *affinis populations*. (a) Historic mutation-scaled migration rates (*M* = *m*/μ) approximately 4*N*_*e*_ generations in the past calculated with Migrate-n. (b) Contemporary migration rates (i.e., *m*) over the last 25–35 years estimated with Bayesass. The thickness of the arrows is proportional to *M* and *m* respectively. Only migration rates significantly different from zero are shown in both panels.

The historical migration estimates (M, hereafter) ranged from 0 to 59.6. Of the 55 pairwise estimates of M for all sets of populations, 32 were significantly different (non-overlapping 95% CI for pairwise migration). The number of migrants per generation, N_em_ (product of theta and M divided by four) ranged from 0 to 16.6. Multiple runs of BAYESASS revealed low contemporary immigration with the exception of CJ, AG, GB, and PL. Our analyses also reveal a source-sink relationship where CJ receives a substantial proportion of migrants from CA (m = 0.13 ± 0.05), while the expected proportion of migrants into CA from CJ is much smaller (m = 0.01 ± 0.01). RC also constitutes a source population for AG, PL, and GB, where these three populations all receive similar proportions of migrants from RC (0.19 ± 0.03, 0.20 ± 0.03 and 0.14 ± 0.04, respectively). Historical and contemporary migration estimates were not significantly correlated (r = 0.02, P < 0.88). There is a contemporary decrease in the migration between populations adjacent to the Panama Canal and the eastern populations (Fig 4).

### Test of population history

The long-term gene flow population model was much more strongly supported than the pure drift model (Prob. of drift model = 0.00, Prob. of gene flow model = 1.00). This indicates that populations have been at drift-migration equilibrium for a substantial period of time. Overall, the effect of genetic drift was low in most of *M*. *affinis*’ populations, with F values ranging from 0.04 to 0.22. ST and CA populations were the most affected by genetic drift (*F* = 0.14 and 0.22, respectively). Estimates of θ from MIGRATE were not correlated with F values from the 2MOD analysis (*r*_*p*_ = -0.05, *P* = 0.89).

## Discussion

Given global tropical forest declines and the importance of reforestation, there is increasing interest in the ecology and evolution of pioneer tropical trees. Specifically, due to their short generation times, these trees provide valuable information regarding the impacts of deforestation on both contemporary and historic gene flow processes, with implications for restoration species selection and translocation strategies. Using novel landscape genetic tools, we evaluate the genetic structure of an early successional species, and reveal that populations of *M*. *affinis* maintain moderately-high levels of genetic diversity and low levels of genetic differentiation despite contemporary deforestation patterns. Furthermore, we show that both elevation and geographic distance play a critical role in increasing genetic differentiation between populations. Thus, we highlight the importance of considering both topography and geographic distance when designing reforestation strategies aimed at preserving genetic diversity within tropical pioneer trees.

### Regional genetic structure and gene flow

Our results indicate that *M*. *affinis* exhibits higher levels of within-population genetic diversity and lower levels of among-population genetic differentiation than many early successional plant species across the globe [87], but similar levels of within-population genetic diversity as other tropical pioneer species [88]. Interestingly, the level of among-population genetic differentiation for *M*. *affinis* was lower than the average reported for tropical trees [89], but similar to light-demanding tropical pioneer species with wind-mediated seed dispersal [88]. In addition, our Bayesian clustering analysis shows substantial admixture levels in *M*. *affinis* populations in the study region, suggesting that *M*. *affinis* experiences substantial dispersal across human-altered landscapes [46], leading to weak but significant genetic differentiation among populations. This high level of genetic diversity and low level of genetic differentiation among populations is likely due to the species’ propensity to colonize recently deforested landscapes, leading to increases in the connectivity among populations throughout the region.

The genetic differentiation among *M*. *affinis* populations was significantly explained by two factors, geographic distance and elevation. First, we found a strong signal of isolation by distance across our study region, as documented for a number of tropical tree species [17,18]. Isolation by distance is frequently stronger in plants dispersed by animals with restricted mobility, such as understory tropical trees, which often have fleshy fruits that are dispersed by small-bodied birds [90]. Previous studies have reported shorter dispersal distances and a lower abundance of long-distance dispersal events for small bird species relative to larger-bodied species [91]. Because *Miconia* seeds are often dispersed by small-bodied bird species [46,92], the strong isolation by distance documented in this study likely reflects the restricted ability of these small-bodied animals to cover the long distances between populations.

We also detected significant isolation by elevation, suggesting that high-elevation populations of *M*. *affinis* are experiencing more restricted gene flow. These results, in line with other recent studies, document a relevant role of elevation as a factor influencing genetic differentiation in plant populations [21,30,93]. Harsher environmental conditions (e.g., lower temperature and higher precipitation) in high-elevation sites compared to low-elevation sites may affect flying conditions for insects and potentially impact pollen-mediated gene flow [25–27]. For instance, lower temperature and higher precipitation severely reduced the pollination service in high-elevation populations of another melastome, *Tibouchina pulchra* [94]. Additionally, elevation can result in reproductive isolation due to phenological shifts that cause temporal separation in the timing of flowering [28]. Specifically, our field research has revealed that populations located at the highest elevation, where there is an earlier onset of the first rains, exhibit a more advanced flowering and fruiting phenology than populations at the lowest elevations (A.C., personal observation), potentially making high-elevation populations more asynchronous in flowering and fruiting relative to other populations. This relationship between reproductive phenology and water availability during the dry season has been reported for other *Miconia* species [95] and may underlie reproductive isolation. This relationship could be strengthened by the dispersal patterns of frugivorous birds, given that understory fruit-eating birds often track changes in fruit abundance across altitudinal gradients within a single mountain side, exploiting higher elevation sites first and lowland sites later [96], potentially enhancing localized gene flow for each temporal fruiting period.

We did not find evidence of increased genetic differentiation with increased deforestation between *M*. *affinis* populations. While forest fragmentation is theorized to drive genetic differentiation among tropical tree populations [32,35], empirical support of this theory is still scarce. Recent reviews suggest that the genetic signature of deforestation may be more evident in younger age-classes or progenies sired in disturbed landscapes ([35] and references therein). Despite the short generation time and relatively young estimated age of the trees sampled in our focal populations, we found no evidence of genetic structuring in response to recent deforestation for *M*. *affinis*. One potential explanation is that the high dispersal and colonization ability of the study species promotes gene flow for *M*. *affinis* across the study region, regardless of forest cover. Past studies have documented that moderate levels of deforestation, whether human-induced or natural, can be well tolerated by disturbance-adapted tree species [97]. As documented in other studies, forest fragment boundaries may not represent mating boundaries due to long-distance dispersal events connecting populations [34]. Our results likewise suggest that the human-altered landscapes along the Panama Canal do not limit gene flow between isolated forest populations.

Other alternative explanations for the genetic structure observed in *M*. *affinis* populations could be related to the construction of the Panama Canal. The Panama Canal was completed in 1914 and led to extensive deforestation in the adjacent areas, except for Barro Colorado Island which, though disturbed, retained substantial old-growth forest [45]. Also, the forests east of Lake Alajuela were primarily undisturbed and retained large areas of near pristine old-growth forest. Our STRUCTURE analyses revealed substantial support for the clustering of *M*. *affinis* populations adjacent to the Panama Canal, excluding the Barro Colorado Island population. Furthermore, our estimates of historical and contemporary migration support a recent collapse of gene flow between populations located east of Lake Alajuela and populations in the Panama Canal Zone. These two analyses provide evidence that deforestation during the construction of the canal may have restricted gene flow; interestingly recovery of the forest in the past 100 years could be preventing detection of a relationship between current deforestation patterns and genetic differentiation.

### Implications for reforestation

In the past 50 years, the Republic of Panama has lost more than 30% of its forested area [45]. This high rate of deforestation is associated with high levels of erosion and sediment deposition in streams and canals. Given the socio-economic value of the Panama Canal and the large amount of water required for shipping traffic, there is great need to reduce sediment deposition and improve the water-holding capacity of the Panama Canal watershed via reforestation [44,98]. To date, however, the majority of the region’s reforestation programs are dominated by fast-growing, exotic timber species, which support only low levels of plant diversity, often promote soil erosion, and provide only limited goods and services to local landholders [5]. Major efforts are being made in Panama to develop reforestation programs with native species that dually increase biodiversity and contribute to rural livelihoods [5], and recent studies have revealed that Panamanian rural farmers are highly interested in land restoration that promotes native trees [6].

However, one of the constraints to using native species in reforestation programs is the lack of ecological information about these species [99]. In this regard, the reproductive processes of tropical trees are particularly important to consider for the long-term viability of the restored population. Our study is one of the first to analyze the gene flow of an early successional tropical tree species using a spatially-explicit landscape genetics approach. Based on our results for *M*. *affinis*, we recommend the use of local genotypes for small-scale restoration, as they may maintain local phenological patterns and promote local adaptation to future land use and climate conditions. We also suggest utilizing species like *M*. *affinis* that demonstrate substantial gene flow at regional-scales, even across large tracts of deforested land. Finally, our results suggest prioritizing the conservation of geographically isolated or high-elevation populations, as these populations may face the greatest challenges of maintaining effective pollen- and seed-mediated gene flow.

## Supporting Information

S1 Fig Correlogram of the initial set of explanatory variables used the MLPE models (Geographic RD, Elevation RD, Forest Cover CD, Precipitation RD, and Temperature RD). The color in both the filled portion of the pie and the shade squares indicates the sign of the correlation, with positive and negative values encoded by blue and red respectively. The intensity of the color increases uniformly as the correlation values move away from 0. There is a significant positive correlation between precipitation and elevation (*r*_*p*_ = 0.51, *P* < 0.05) and a marginally significant positive correlation between mean annual temperature and elevation (*r*_*p*_ = 0.42, *P* = 0.08), thus we opted to include only Elevation RD in our models.
(TIFF)

S2 Fig Magnitude of ΔK as a function of K over 10 runs of STRUCTURE for each K value from 1 to 11. Runs used a total number of iterations of 150,000 with a burn-in of 50,000. The admixture and the correlated frequency models were used.
(TIF)

S3 Fig Genetic structure of *M*. *affinis* populations based on STRUCTURE output for K = 3. (a) Structure bar plot with individuals sorted by populations. Each vertical bar represents a single individual and color segments are proportional to its membership in three genetic clusters inferred from STRUCTURE analyses. From left to right, populations are sorted by increasing distance to the Panama Canal. (b) Populations represented by pie charts with the mean proportion of membership of each population for the inferred number of K = 3 genetic groups. In the background, higher elevation is represented by darker shades (0–940 m.a.s.l.).
(TIF)

S1 File Genotypes of *M*. *affinis* trees in the eleven study populations. (XLSX)

S1 Table Predicted lifespan for *M*. *affinis*. DBH_max_ refers the largest DBH found for a tree in each of the five intensively surveyed populations. Predicted lifespan was calculated dividing DBH_max_ by the mean annual growth of *M*. *affinis* in the 50-ha BCI plot (i.e., 1.75 mm per year).
(DOCX)

S2 Table Model averaged coefficients (β) and their standard errors (SE) calculated from the candidate model set using allelic richness as the response variable (i.e. models with ΔAIC < 5). Spatial isolation refers the mean geographic distance of each population to the other populations. Forest cover represents the percentage of forest cover within a 1, 2 and 3 km radius. Forest cover considers all vegetation taller than 5 meters in height. Model averaged coefficients not overlapping with zero are shown in bold.
(DOCX)

S3 Table Model averaged coefficients (β) and their standard errors (SE) calculated from the candidate model set using allelic private richness as the response variable (i.e. models with ΔAIC < 5). Spatial isolation refers the mean geographic distance of each population to the other populations. Forest cover represents the percentage of forest cover within a 1, 2, and 3 km radius. Forest cover considers all vegetation taller than 5 meters in height. Model averaged coefficients not overlapping with zero are indicated with asterisks.
(DOCX)

S4 Table Model averaged coefficients (β) and their standard errors (SE) calculated from the candidate model set using unbiased Nei’s gene diversity as the response variable (i.e. models with ΔAIC < 5). Spatial isolation refers the mean geographic distance of each population to the other populations. Forest cover represents the percentage of forest cover within a 1, 2, and 3 km radius. Forest cover considers all vegetation taller than 5 meters in height.
(DOCX)

S5 Table Ranked models explaining landscape effects on genetic differentiation (i.e., G_ST_) among *Miconia affinis*’ populations in Panama. Models are ranked based upon the difference between Akaike’s Information Criterion (AIC) in each individual model and the lowest AIC model (ΔAIC). Akaike’s weights (ω_i_) for each model are listed. The genetic differentiation between populations was calculated as [G_ST_ / (1-G_ST_)]. Geographic refers to log-transformed null resistance distance.
(DOCX)

S6 Table Model averaged coefficients (β) and their standard errors (SE) calculated from the candidate model set (i.e. models with ΔAIC < 5). Model averaged coefficients not overlapping with zero are indicated with asterisks. The genetic differentiation between populations was calculated as [G_ST_ / (1-G_ST_)]. Geographic refers to log-transformed null resistance distance.
(DOCX)

S7 Table Ranked models explaining landscape effects on genetic differentiation (i.e., D_ST_) among *Miconia affinis*’ populations in Panama. Models are ranked based upon the difference between Akaike’s Information Criterion (AIC) in each individual model and the lowest AIC model (ΔAIC). Akaike’s weights (ω_i_) for each model are listed. The genetic differentiation between populations was calculated as [D_ST_ / (1-D_ST_)]. Geographic refers to log-transformed null resistance distance.
(DOCX)

S8 Table Model averaged coefficients (β) and their standard errors (SE) calculated from the candidate model set (i.e. models with ΔAIC < 5). Model averaged coefficients not overlapping with zero are indicated with asterisks. The genetic differentiation between populations was calculated as [D_ST_ / (1-D_ST_)]. Geographic refers to log-transformed null resistance distance.
(DOCX)

S9 Table Ranked models explaining landscape effects on F_ST_ between *Miconia affinis*’ among populations in Panama. Models are ranked based upon the difference between Akaike’s Information Criterion (AIC) in each individual model and the lowest AIC model (ΔAIC). Akaike’s weights (ω_i_) provide the weight for each model. Elevation was computed as the Euclidean elevation distance matrix. Deforestation represents the percentage of deforested cover and was coded as a resistance variable. Geographic refers to null resistance distance.
(DOCX)

S10 Table Model averaged coefficients (β) and their standard errors (SE) calculated from the candidate model set (i.e. models with ΔAIC < 5). Model averaged coefficients not overlapping with zero are indicated with asterisks. Population pairwise F_ST_ were used as estimates of genetic differentiation between *M*. *affinis*’ populations. Elevation was computed as the Euclidean elevation distance matrix. Deforestation refers to the percentage of deforested cover and was coded as a resistance variable. Geographic refers to null resistance distance. All explanatory variables are centered.
(DOCX)
