# Supplementary figures and images for: Elevation, Not Deforestation, Promotes Genetic Differentiation in a Pioneer Tropical Tree
Source: PLoS One. 2016 Jun 9;11(6):e0156694. doi: 10.1371/journal.pone.0156694 (PMC4900633; doi:10.1371/journal.pone.0156694)

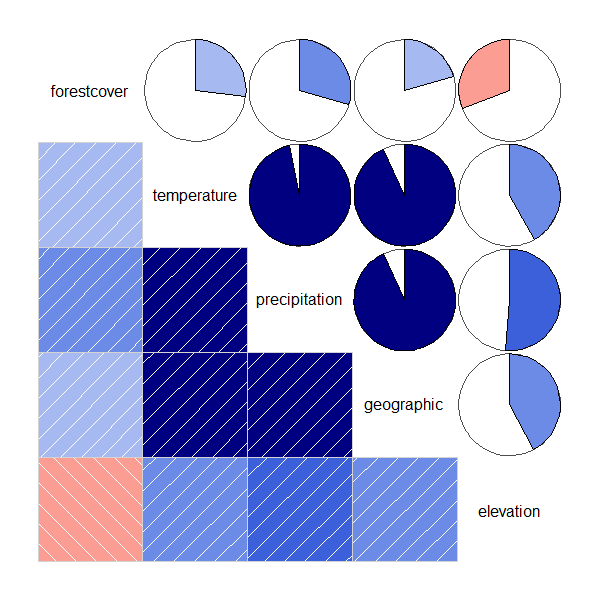

Supplement: S1 Fig — The color in both the filled portion of the pie and the shade squares indicates the sign of the correlation, with positive and negative values encoded by blue and red respectively. The intensity of the color increases uniformly as the correlation values move away from 0. There is a significant positive correlation between precipitation and elevation (rp = 0.51, P < 0.05) and a marginally significant positive correlation between mean annual temperature and elevation (rp = 0.42, P = 0.08), thus we opted to include only Elevation RD in our models. (TIFF) [file pone.0156694.s001.tiff]

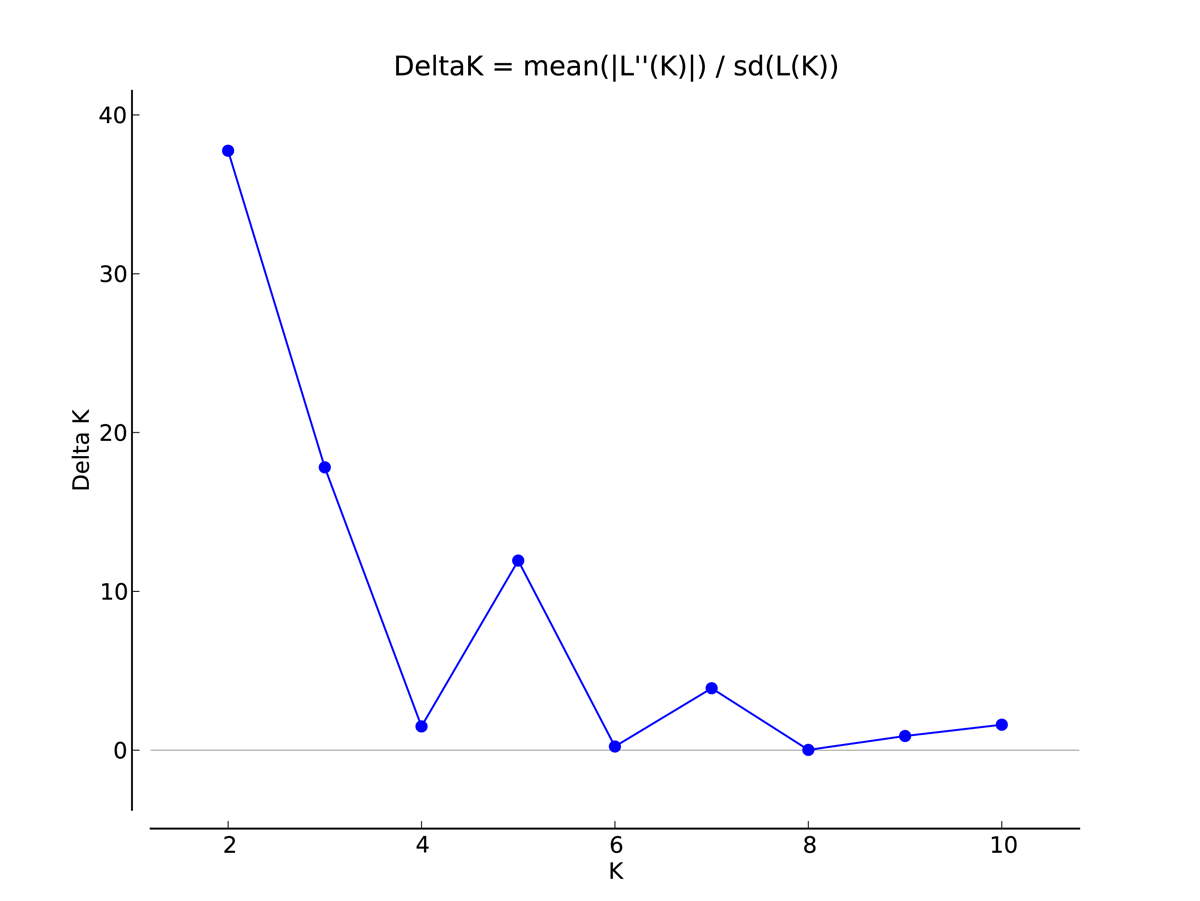

Supplement: S2 Fig — Runs used a total number of iterations of 150,000 with a burn-in of 50,000. The admixture and the correlated frequency models were used. (TIF) [file pone.0156694.s002.tif]

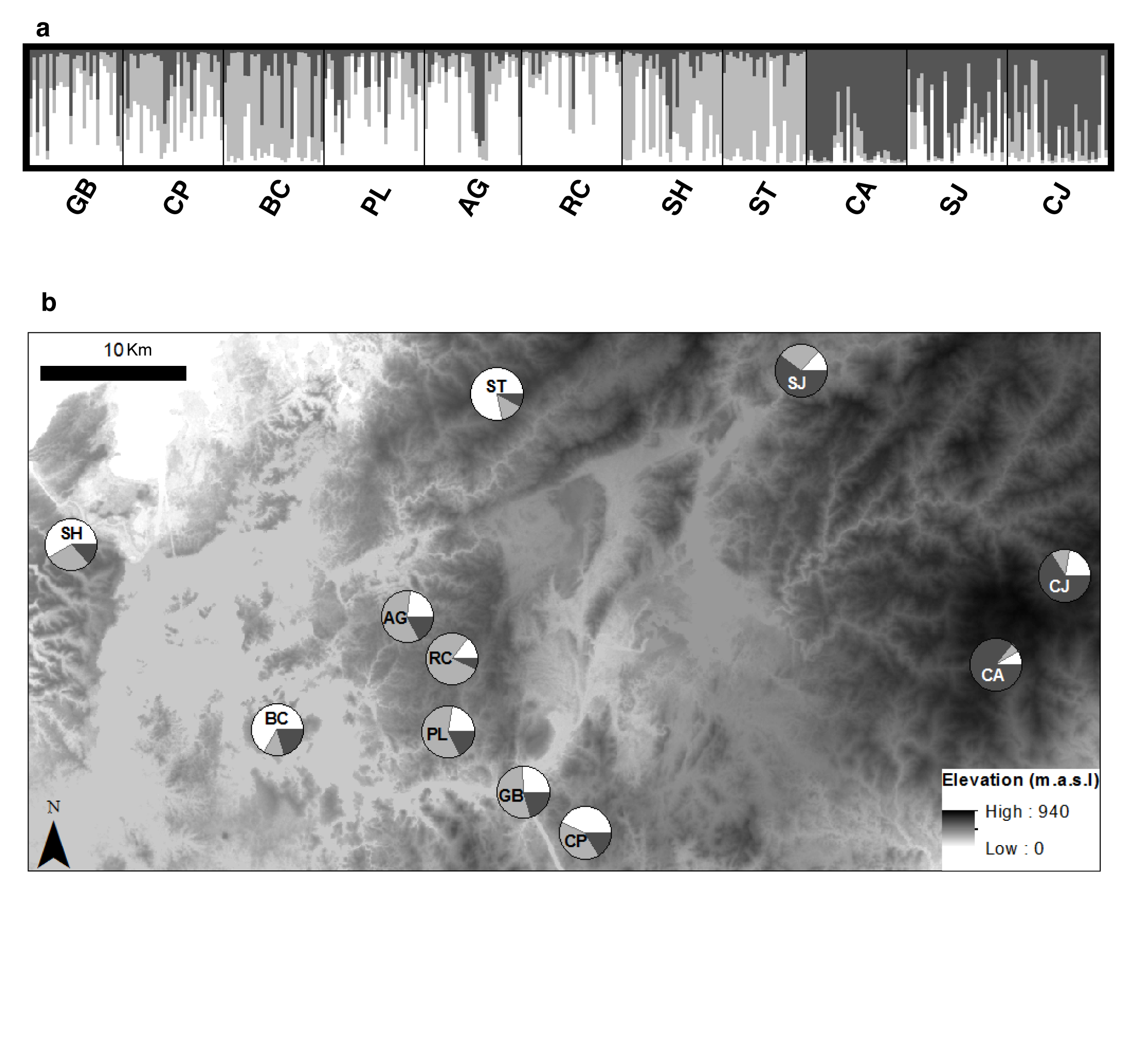

Supplement: S3 Fig — (a) Structure bar plot with individuals sorted by populations. Each vertical bar represents a single individual and color segments are proportional to its membership in three genetic clusters inferred from STRUCTURE analyses. From left to right, populations are sorted by increasing distance to the Panama Canal. (b) Populations represented by pie charts with the mean proportion of membership of each population for the inferred number of K = 3 genetic groups. In the background, higher elevation is represented by darker shades (0–940 m.a.s.l.). (TIF) [file pone.0156694.s003.tif]
