# Supplementary material for: Elevation, Not Deforestation, Promotes Genetic Differentiation in a Pioneer Tropical Tree
Source: PLoS One. 2016 Jun 9;11(6):e0156694. doi: 10.1371/journal.pone.0156694 (PMC4900633; doi:10.1371/journal.pone.0156694)
Supplement: S1 Table — DBHmax refers the largest DBH found for a tree in each of the five intensively surveyed populations. Predicted lifespan was calculated dividing DBHmax by the mean annual growth of M. affinis in the 50-ha BCI plot (i.e., 1.75 mm per year). (DOCX) [file pone.0156694.s005.docx]

| Population | DBH_max_ (mm) | Predicted lifespan (years) |
| --- | --- | --- |
| CP | 117.7 | 67.3 |
| GB | 125.5 | 71.7 |
| PL | 109.3 | 62.3 |
| RC | 95.3 | 54.5 |
| AG | 115.3 | 65.9 |
| *mean* | *112.6* | *64.3* |

**S1 Table. Predicted lifespan for *M. affinis*.** DBH_max_ refers the largest DBH found for a tree in each of the five intensively surveyed populations. Predicted lifespan was calculated dividing DBH_max_ by the mean annual growth of *M. affinis* in the 50-ha BCI plot (i.e., 1.75 mm per year).
