# Supplementary material for: Elevation, Not Deforestation, Promotes Genetic Differentiation in a Pioneer Tropical Tree
Source: PLoS One. 2016 Jun 9;11(6):e0156694. doi: 10.1371/journal.pone.0156694 (PMC4900633; doi:10.1371/journal.pone.0156694)
Supplement: S2 Table — Spatial isolation refers the mean geographic distance of each population to the other populations. Forest cover represents the percentage of forest cover within a 1, 2 and 3 km radius. Forest cover considers all vegetation taller than 5 meters in height. Model averaged coefficients not overlapping with zero are shown in bold. (DOCX) [file pone.0156694.s006.docx]

**S2 Table. Model averaged coefficients (β) and their standard errors (SE) calculated from the candidate model set using allelic richness as the response variable (i.e. models with ΔAIC < 5).** Spatial isolation refers the mean geographic distance of each population to the other populations. Forest cover represents the percentage of forest cover within a 1, 2 and 3 km radius. Forest cover considers all vegetation taller than 5 meters in height.

|  |  |  | | Allelic richness | | | | | | | | | | | |
| --- | --- | --- | --- | --- | --- | --- | --- | --- | --- | --- | --- | --- | --- | --- | --- |
|  | Forest cover (1 km) | | | | |  | Forest cover (2 km) | | | |  | Forest cover (3 km) | | | |
|  | β | SE | Z value | | P value |  | β | SE | Z value | P value |  | β | SE | Z value | P value |
| Spatial isolation | -0.00296 | 0.01392 | 0.186 | | 0.85 |  | -0.00295 | 0.01393 | 0.186 | 0.85 |  | -0.00294 | 0.01391 | 0.185 | 0.85 |
| Elevation | -0.00044 | 0.00035 | 1.106 | | 0.27 |  | -0.00044 | 0.00035 | 1.105 | 0.27 |  | -0.00044 | 0.00035 | 1.104 | 0.27 |
| Forest cover | 0.00004 | 0.00546 | 0.006 | | 0.99 |  | -0.00068 | 0.00931 | 0.063 | 0.95 |  | -0.00110 | 0.00803 | 0.119 | 0.91 |
