# Supplementary material for: Elevation, Not Deforestation, Promotes Genetic Differentiation in a Pioneer Tropical Tree
Source: PLoS One. 2016 Jun 9;11(6):e0156694. doi: 10.1371/journal.pone.0156694 (PMC4900633; doi:10.1371/journal.pone.0156694)
Supplement: S3 Table — Spatial isolation refers the mean geographic distance of each population to the other populations. Forest cover represents the percentage of forest cover within a 1, 2, and 3 km radius. Forest cover considers all vegetation taller than 5 meters in height. Model averaged coefficients not overlapping with zero are indicated with asterisks. (DOCX) [file pone.0156694.s007.docx]

**S3 Table. Model averaged coefficients (β) and their standard errors (SE) calculated from the candidate model set using allelic private richness as the response variable (i.e. models with ΔAIC < 5).** Spatial isolation refers the mean geographic distance of each population to the other populations. Forest cover represents the percentage of forest cover within a 1, 2 and 3 km radius. Forest cover considers all vegetation taller than 5 meters in height.

|  | | | | | | | | | | | | | | | |
| --- | --- | --- | --- | --- | --- | --- | --- | --- | --- | --- | --- | --- | --- | --- | --- |
|  |  |  | | Allelic private richness | | | | | | | | | | | |
|  | Forest cover (1 km) | | | | |  | Forest cover (2 km) | | | |  | Forest cover (3 km) | | | |
|  | β | SE | Z value | | P value |  | β | SE | Z value | P value |  | β | SE | Z value | P value |
| Spatial isolation | 0.00224 | 0.00232 | 0.833 | | 0.40 |  | 0.00222 | 0.00232 | 0.826 | 0.41 |  | 0.00269 | 0.00125 | 1.863 | 0.06* |
| Elevation | 0.00006 | 0.00007 | 0.702 | | 0.48 |  | 0.00150 | 0.00171 | 0.757 | 0.45 |  | 0.00206 | 0.00218 | 0.814 | 0.42 |
| Forest cover | 0.00049 | 0.00103 | 0.414 | | 0.68 |  | 0.00006 | 0.00007 | 0.697 | 0.49 |  | 0.00005 | 0.00006 | 0.683 | 0.49 |

*Model averaged coefficients not overlapping with zero.
