# Supplementary material for: Elevation, Not Deforestation, Promotes Genetic Differentiation in a Pioneer Tropical Tree
Source: PLoS One. 2016 Jun 9;11(6):e0156694. doi: 10.1371/journal.pone.0156694 (PMC4900633; doi:10.1371/journal.pone.0156694)
Supplement: S4 Table — Spatial isolation refers the mean geographic distance of each population to the other populations. Forest cover represents the percentage of forest cover within a 1, 2, and 3 km radius. Forest cover considers all vegetation taller than 5 meters in height. (DOCX) [file pone.0156694.s008.docx]

**S4 Table. Model averaged coefficients (β) and their standard errors (SE) calculated from the candidate model set using unbiased Nei’s gene diversity as the response variable (i.e. models with ΔAIC < 5).** Spatial isolation refers the mean geographic distance of each population to the other populations. Forest cover represents the percentage of forest cover within a 1, 2 and 3 km radius. Forest cover considers all vegetation taller than 5 meters in height.

|  |  |  | | Nei’s gene diversity | | | | | | | | | | | |
| --- | --- | --- | --- | --- | --- | --- | --- | --- | --- | --- | --- | --- | --- | --- | --- |
|  | Forest cover (1 km) | | | | |  | Forest cover (2 km) | | | |  | Forest cover (3 km) | | | |
|  | β | SE | Z value | | P value |  | β | SE | Z value | P value |  | β | SE | Z value | P value |
| Spatial isolation | -0.00008 | 0.00004 | 1.513 | | 0.13 |  | -0.00008 | 0.00004 | 1.510 | 0.13 |  | -0.00008 | 0.00004 | 1.508 | 0.13 |
| Elevation | -0.00212 | 0.00170 | 1.082 | | 0.28 |  | -0.00211 | 0.00170 | 1.081 | 0.28 |  | -0.00211 | 0.00170 | 1.079 | 0.28 |
| Forest cover | 0.00006 | 0.00075 | 0.067 | | 0.95 |  | -0.00009 | 0.00128 | 0.064 | 0.95 |  | -0.00051 | 0.00109 | 0.402 | 0.69 |
