# Supplementary material for: Elevation, Not Deforestation, Promotes Genetic Differentiation in a Pioneer Tropical Tree
Source: PLoS One. 2016 Jun 9;11(6):e0156694. doi: 10.1371/journal.pone.0156694 (PMC4900633; doi:10.1371/journal.pone.0156694)
Supplement: S6 Table — Model averaged coefficients not overlapping with zero are indicated with asterisks. The genetic differentiation between populations was calculated as [GST / (1-GST)]. Geographic refers to log-transformed null resistance distance. (DOCX) [file pone.0156694.s010.docx]

**S6 Table. Model averaged coefficients (β) and their standard errors (SE) calculated from the candidate model set (i.e. models with ΔAIC < 5).** The genetic differentiation between populations was calculated as [G_ST_ / (1-G_ST_)]. Geographic refers to log-transformed null resistance distance.

|  | β | SE | Z value | P value |
| --- | --- | --- | --- | --- |
| Geographic | 0.17780 | 0.04660 | 3.732 | 0.001* |
| Elevation | 0.00006 | 0.00003 | 2.369 | 0.018* |
| Deforestation | -0.00059 | 0.00130 | 0.446 | 0.656 |

***** Model averaged coefficients not overlapping with zero.
