# Supplementary material for: Elevation, Not Deforestation, Promotes Genetic Differentiation in a Pioneer Tropical Tree
Source: PLoS One. 2016 Jun 9;11(6):e0156694. doi: 10.1371/journal.pone.0156694 (PMC4900633; doi:10.1371/journal.pone.0156694)
Supplement: S7 Table — Models are ranked based upon the difference between Akaike’s Information Criterion (AIC) in each individual model and the lowest AIC model (ΔAIC). Akaike’s weights (ωi) for each model are listed. The genetic differentiation between populations was calculated as [DST / (1-DST)]. Geographic refers to log-transformed null resistance distance. (DOCX) [file pone.0156694.s011.docx]

**S7 Table. Ranked models explaining landscape effects on genetic differentiation (i.e., D_ST_) among *Miconia affinis*’ populations in Panama.** Models are ranked based upon the difference between Akaike’s Information Criterion (AIC) ranked ith and the top-ranked model (ΔAIC). Akaike’s weights (ω_i_) for each model are listed. The genetic differentiation between populations was calculated as [D_ST_ / (1-D_ST_)]. Geographic refers to log-transformed null resistance distance.

| Model statement | AIC | ΔAIC | ω_i_ |
| --- | --- | --- | --- |
| Geographic + Elevation | -169.51 | 0.00 | 0.432 |
| Geographic + Elevation + Deforestation | -168.40 | 1.10 | 0.249 |
| Geographic | -168.13 | 1.37 | 0.217 |
| Geographic + Deforestation | -166.54 | 2.97 | 0.098 |
| Elevation | -159.52 | 9.98 | 0.003 |
| Elevation + Deforestation | -157.52 | 11.98 | 0.001 |
| Deforestation | -150.46 | 19.04 | 0.000 |
