# Supplementary material for: Elevation, Not Deforestation, Promotes Genetic Differentiation in a Pioneer Tropical Tree
Source: PLoS One. 2016 Jun 9;11(6):e0156694. doi: 10.1371/journal.pone.0156694 (PMC4900633; doi:10.1371/journal.pone.0156694)
Supplement: S8 Table — Model averaged coefficients not overlapping with zero are indicated with asterisks. The genetic differentiation between populations was calculated as [DST / (1-DST)]. Geographic refers to log-transformed null resistance distance. (DOCX) [file pone.0156694.s012.docx]

**S8 Table. Model averaged coefficients (β) and their standard errors (SE) calculated from the candidate model set (i.e. models with ΔAIC < 5).** The genetic differentiation between populations was calculated as [D_ST_ / (1-D_ST_)]. Geographic refers to log-transformed null resistance distance.

|  | β | SE | Z value | P value |
| --- | --- | --- | --- | --- |
| Geographic | 0.6890 | 0.1800 | 3.748 | 0.001* |
| Elevation | 0.0002 | 0.0001 | 1.927 | 0.053* |
| Deforestation | 0.0038 | 0.0045 | 0.818 | 0.414 |

***** Model averaged coefficients not overlapping with zero.
