# Supplementary material for: Elevation, Not Deforestation, Promotes Genetic Differentiation in a Pioneer Tropical Tree
Source: PLoS One. 2016 Jun 9;11(6):e0156694. doi: 10.1371/journal.pone.0156694 (PMC4900633; doi:10.1371/journal.pone.0156694)
Supplement: S9 Table — Models are ranked based upon the difference between Akaike’s Information Criterion (AIC) in each individual model and the lowest AIC model (ΔAIC). Akaike’s weights (ωi) provide the weight for each model. Elevation was computed as the Euclidean elevation distance matrix. Deforestation represents the percentage of deforested cover and was coded as a resistance variable. Geographic refers to null resistance distance. (DOCX) [file pone.0156694.s013.docx]

**S9 Table. Ranked models explaining landscape effects on F_ST_ between *Miconia affinis*’ among populations in Panama.** Models are ranked based upon the difference between Akaike’s Information Criterion (AIC) ranked ith and the top-ranked model (ΔAIC). Akaike’s weights (ω_i_) provide the weight for each model. Elevation was computed as the Euclidean elevation distance matrix. Deforestation represents the percentage of deforested cover and was coded as a resistance variable. Geographic refers to null resistance distance.

| Model statement | AIC | ΔAIC | ω_i_ |
| --- | --- | --- | --- |
| Geographic + Elevation | -241.03 | 0.00 | 0.622 |
| Geographic + Elevation + Deforestation | -239.05 | 1.98 | 0.231 |
| Geographic | -237.49 | 3.55 | 0.106 |
| Geographic + Deforestation | -235.51 | 5.52 | 0.039 |
| Elevation | -228.13 | 12.91 | 0.001 |
| Elevation + Deforestation | -227.93 | 13.10 | 0.001 |
| Deforestation | -216.16 | 24.87 | 0.000 |
