# Supplementary material for: Elevation, Not Deforestation, Promotes Genetic Differentiation in a Pioneer Tropical Tree
Source: PLoS One. 2016 Jun 9;11(6):e0156694. doi: 10.1371/journal.pone.0156694 (PMC4900633; doi:10.1371/journal.pone.0156694)
Supplement: S10 Table — Model averaged coefficients not overlapping with zero are indicated with asterisks. Population pairwise FST were used as estimates of genetic differentiation between M. affinis’ populations. Elevation was computed as the Euclidean elevation distance matrix. Deforestation refers to the percentage of deforested cover and was coded as a resistance variable. Geographic refers to null resistance distance. All explanatory variables are centered. (DOCX) [file pone.0156694.s014.docx]

**S10 Table. Model averaged coefficients (β) and their standard errors (SE) calculated from the candidate model set (i.e. models with ΔAIC < 5).** Population pairwise F_ST_ were used as estimates of genetic differentiation between *M. affinis*’ populations. Elevation was computed as the Euclidean elevation distance matrix. Deforestation refers to the percentage of deforested cover and was coded as a resistance variable. Geographic refers to null resistance distance. All explanatory variables are centered.

|  | β | SE | Z value | P value |
| --- | --- | --- | --- | --- |
| Geographic | 0.37960 | 0.09664 | 3.845 | <0.001* |
| Elevation | 0.00005 | 0.00002 | 2.29 | 0.022* |
| Forest cover | -0.00044 | 0.00322 | 0.135 | 0.893 |

*Model averaged coefficients not overlapping with zero.
